# Preparation of Sodium Silicon-Modified Maize Stalk Biochar-Based Fertilizer and Its Impacts on Foxtail Millet Growth and Soil Properties

**DOI:** 10.3390/plants15152364

**Published:** 2026-07-31

**Authors:** Xue Gao, Ruihua Han, Feiyu Liu, Chenyang Wang, Yanyan Duan, Huiling Du, Shuqi Dong, Chunyan Hu

**Affiliations:** 1 College of Agriculture, Shanxi Agriculture University, Jinzhong 030801, China; gao_xue1020@163.com (X.G.); 15148745002@163.com (R.H.); 13323049979@163.com (F.L.); ww1286687680@163.com (C.W.); 17835714706@163.com (Y.D.); dong-s-q@163.com (S.D.); hucy4216@sxau.edu.cn (C.H.); 2 Shanxi Institute of Functional Agriculture, Shanxi Agricultural University, Jinzhong 030801, China

**Keywords:** foxtail millet, sodium silicate modification, biochar-based fertilizer, soil properties, delayed release system for nutrients

## Abstract

There exist multiple conflicting challenges in dryland agricultural production: excessive chemical fertilizer input contrasts with low utilization efficiency of maize straw resources, and long-term over-fertilization triggers continuous degradation of farmland soil fertility. To explore the soil improvement and growth-promoting potential of silicon-modified biochar-based fertilizers, this study conducted relevant preparation and pot experiments. This study used maize stover as feedstock to investigate the effects of pyrolysis temperature and silica modification on the structural characteristics of biochar. Raw biochar was pyrolyzed at 400–600 °C and subsequently modified with sodium silicate. Biochar-based fertilizers were then produced using modified biochar as the carrier, and their influences on soil physicochemical properties, soil enzyme activities, and foxtail millet growth were analyzed via pot experiments. The results indicate that 500 °C is the optimal pyrolysis temperature for maize straw biochar. After sodium silicate modification, the specific surface area of modified biochar increased by 18.84% relative to unmodified raw biochar, accompanied by a more developed surface pore structure. The application of biochar-based fertilizers significantly reduced soil pH and increased soil carbon stocks and available nutrient content. All biochar-based fertilizer treatments significantly improved foxtail millet growth, among which the BF3 treatment showed the strongest growth-promoting performance. Compared with the sole chemical fertilizer (CF), BF3 increased plant height by 63.72% and total biomass by 54.27%, while stem diameter decreased by 11.41%. In summary, sodium silicate-modified biochar-based fertilizer pyrolyzed at 500 °C can effectively improve soil quality and promote millet growth, with a biochar-to-fertilizer ratio of 1:3 being the optimal formulation for dryland millet cultivation. This study provides a direct basis for the compatibility evaluation of biochar-based fertilizers in dryland foxtail millet cultivation.

## 1. Introduction

Foxtail millet is a vital minor grain crop in dry farming regions across northern China [1]. Shanxi Province ranks among the major foxtail millet producing areas with a long cultivation history. It is characterized by strong tolerance to poor soil fertility, drought resistance and wide adaptability, and plays a pivotal role in advancing sustainable agriculture and safeguarding regional food security [2]. Crop yield serves as the core evaluation index in agricultural production, and high yields depend on scientific fertilization regimes and high soil fertility [3]. Nevertheless, excessive chemical fertilizer application not only reduced planting efficiency and caused fertilizer waste, but also exacerbates environmental issues including soil compaction, acidification and salinization [4]. Therefore, exploring green fertilization technologies that simultaneously enhance soil quality and crop productivity is of great significance for promoting the sustainable development of the foxtail millet industry and alleviating the ecological degradation of farmland. 

Biochar refers to carbon-rich, highly aromatic solid organic substances generated by high-temperature pyrolysis of agricultural wastes such as straw and branches under oxygen-free or oxygen-limited conditions [5]. Benefiting from its unique pore structure, large specific surface area and abundant surface functional groups, biochar possesses broad application prospects in soil remediation, carbon sequestration and pollution abatement. Previous studies verified biochar as an effective soil amendment rich in various mineral elements [6]. After being incorporated into soil, biochar improved soil physical structure, activated soil microbial activity [7], optimized nutrient cycling in the crop-soil system, and consequently facilitated crop growth and yield formation [8,9]. Pyrolysis temperature directly regulated the physicochemical properties of biochar. As pyrolysis temperature increased, the specific surface area and porosity of biochar increased remarkably, oxygen-containing functional groups decomposed gradually, and aromaticity rose sharply [10]. However, biochar itself contains limited nutrients. To maximize its agricultural application potential, numerous researchers have compounded biochar with chemical fertilizers to manufacture biochar-based fertilizers. The pores and functional groups of biochar immobilized soil nutrients to achieve slow fertilizer release, effectively improving fertilizer use efficiency, reducing nutrient loss and ultimately boosting crop yields [11]. Shabbir et al. [12] found that biochar can alleviate drought stress on corn and enhance plant resilience. Ashenafi et al. [13] reported that biochar-based fertilizers alleviated soil acidification and enhanced soil nutrient retention, raising wheat yield by 49.0% in acidic soils. Dissanayake et al. [14] demonstrated that biochar-based fertilizers effectively lowered planting costs without yield reduction, matching the cultivation demands of fertilizer-intensive crops. 

However, there remains room for further optimization of raw biochar in terms of specific surface area and surface functional group diversity. Post-impregnation modification with sodium silicate is a low-cost approach to enhance the performance of straw-based biochar as a carrier, differing from traditional modification methods that involve pretreatment of biomass feedstocks. This post-treatment enables silicate ions to thoroughly penetrate the well-developed porous structure of the formed biochar, depositing on pore walls or forming fine siliceous microcrystals, thereby increasing the number of mesopores and micropores and significantly enhancing both specific surface area and cation exchange capacity—thus enabling precise modification of biochar’s pore characteristics and surface morphology [15]. Current research indicates that after modification and thorough water washing, the free sodium salts on the surface of biochar can be removed, significantly reducing the sodium input load; at the same time, the extensive pores of silicon-modified charcoal can adsorb and fix the soil exchangeable sodium, increasing the potassium-sodium ratio of the soil, and to a certain extent alleviating the toxicity of sodium ions, achieving a balance between the improvement benefits and the sodium environmental risks [16]. Therefore, conducting post-impregnation silicon modification on corn straw biochar after pyrolysis, and systematically investigating changes in its pore structure and morphology, is a necessary prerequisite for rationally designing functional carbon-based fertilizer carriers.

Post-impregnation modification with sodium silicate generates silanol and siloxane groups on the surface of pyrolyzed biochar pores. These groups fix mineral nutrients such as nitrogen, phosphorus, and potassium through dual mechanisms: proton exchange by silanols and physical retention within pores, thereby delaying nutrient leaching and enabling sustained slow-release supply [17]. Existing studies have confirmed that silicon-modified straw biochar, when used as a fertilizer carrier, can reduce the initial burst release of nutrients [18]. However, most research has focused either on the adsorption properties of the modified materials themselves or on improving paddy soil, while systematic validation regarding the compatibility and effectiveness of silicon-modified carbon-based fertilizers specifically for upland millet cultivation remains lacking [19].

The primary objectives of this study are to investigate the preparation process of charcoal-based fertilizers, their slow-release properties, and their effectiveness on millet growth. Specifically: (1) to examine the effect of pyrolysis temperature on biomass charcoal; (2) to assess the impact of modified biochar on fertilizer slow release; and (3) to evaluate the effects of charcoal-based fertilizers on millet growth and soil properties.

## 2. Results

### 2.1. Effects of Pyrolysis Temperature on Physicochemical Structures of Raw and Silicon-Modified Biochar

#### 2.1.1. Elemental Composition and pH of Biochar

Table 1 shows that the carbon (C) content and carbon-nitrogen ratio (C/N) of both raw and silicon-modified biochar rose with increasing pyrolysis temperature, while hydrogen (H) and oxygen (O) contents presented a descending trend. The carbon content of raw biochar increased from 65.04% at 400 °C to 70.22% at 600 °C. After sodium silicate modification, the C, H and O contents of each biochar sample declined compared with unmodified counterparts, whereas the C/N ratio further increased. Silicon modification reduced sulfur content in all biochar samples. These elemental compositions jointly regulated the surface chemical properties of biochar and determined its adsorption capacity for exogenous ions [20]. All biochar samples were alkaline, with pH values ranging from 9.43 to 10.23. The pH of raw biochar slightly increased with rising pyrolysis temperature, while silicon-modified biochar displayed marginally lower pH values than unmodified biochar produced at the same temperature, which may be explained by silicates introduced via sodium silicate buffering biochar alkalinity.

#### 2.1.2. Pore Structure of Biochar

Table 2 presents the variations in specific surface area, pore size, pore volume and pH value of biochar under different pyrolysis temperatures and silicon modification treatments. The specific surface area of raw biochar first increased and then decreased as pyrolysis temperature rose, peaking at 221.79 m^2^/g under 500 °C pyrolysis. Additionally, silicon modification significantly elevated the specific surface area, which followed a similar temperature-dependent trend and reached a maximum of 262.79 m^2^/g at 500 °C, representing an 18.48% increase relative to BC-500. Pore size exhibited a consistent variation pattern, while pore volume decreased first and then increased with temperature elevation. Modified biochar exhibited larger overall pore sizes yet lower pore volumes, a phenomenon attributed to the attachment and filling of silicon substances inside pores during modification. The enlarged specific surface area and optimized pore structure enhanced biochar’s capacity to adsorb and retain fertilizer nutrients [21].

#### 2.1.3. Surface Morphology Characteristics of Biochar

**Figure 1:**
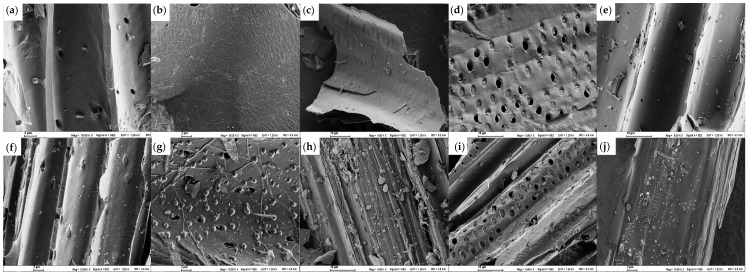
Scanning electron microscopy (SEM) micrographs of maize stover biochar pyrolyzed at 400–600 °C and sodium silicate-modified biochar. (**a**) BC-400; (**b**) BC-450; (**c**) BC-500; (**d**) BC-550; (**e**) BC-600; (**f**) BCSi-400; (**g**) BCSi-450; (**h**) BCSi-500; (**i**) BCSi-550; (**j**) BCSi-600.

Figure 1 displays the SEM images of raw biochar produced at 400–600 °C (Figure 1a–e) and silicon-modified biochar (Figure 1f–j). The micrographs revealed that raw biochar produced at high pyrolysis temperatures possessed smooth surfaces and simple pore structures. In contrast, sodium silicate modification reconstructed the biochar surface into a more complex, uniform pore network with tighter inter-pore connections. Tiny micropores and fragmentary protrusive crystalline structures were observed under high magnification; it can be observed that the surface forms fragmented protrusions, suggesting that siliceous particles derived from silicates are present [22]. This structural characteristic provided abundant effective adsorption sites to strengthen biochar’s capacity to adsorb and immobilize nutrient ions, reduce nutrient leaching loss, and lay a structural foundation for its application as a fertilizer carrier.

#### 2.1.4. Surface Functional Groups of Biochar

**Figure 2:**
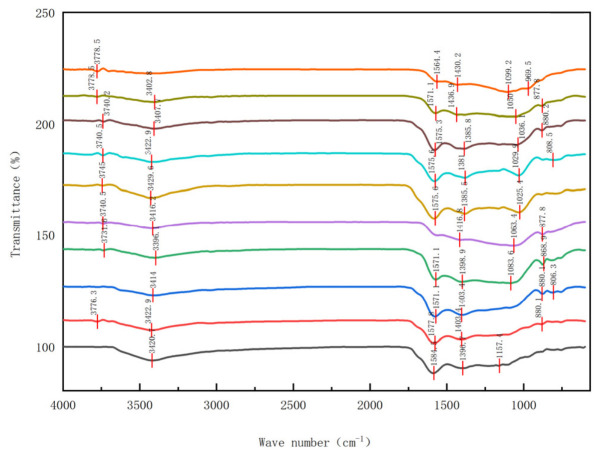
Fourier-transform infrared (FTIR) spectra of raw and silicon-modified maize stover biochar pyrolyzed at 400–600 °C. Spectra from top to bottom correspond to BC-400, BC-450, BC-500, BC-550, BC-600, BCSi-400, BCSi-450, BCSi-500, BCSi-550, BCSi-600, respectively.

Figure 2 presents the Fourier-transform infrared (FTIR) spectra of all biochar samples. For raw biochar, the vibration intensity of the -OH stretching peak near 3400 cm^−1^ gradually weakened as pyrolysis temperature increased, while silicon-modified biochar exhibited stronger -OH peaks than unmodified biochar produced at identical temperatures. The C=C skeleton vibration band near 1580 cm^−1^ intensified with temperature elevation. Silicon-modified biochar showed slightly weaker aromatic C=C peaks than raw biochar at the same temperature, yet no obvious peak shift occurred, indicating silicon modification did not destroy the aromatic carbon skeleton of biochar and only grafted silicon-based functional groups on the surface with limited impacts on carbon frameworks. The C-H bond at 1400 cm^−1^ and C-O bond near 1100 cm^−1^ displayed gradually attenuated vibration intensities on raw biochar with rising pyrolysis temperature. Silicon modification barely altered the absorption intensity of aliphatic C-H bonds at 1400 cm^−1^. A new strong absorption peak emerged near 1100 cm^−1^ on modified biochar, which is attributed to the stretching vibration of the siloxane bond. It is therefore speculated that this may be due to a condensation reaction, resulting in the characteristic vibrational peak of the formed siloxane bond (Si-O). Sodium silicate reacts with oxygen-containing groups on the biochar surface through a condensation reaction, successfully binding the silicon component to the surface of the carbon framework, and the peak intensity slightly increased with higher pyrolysis temperatures. Elevated pyrolysis temperature prompted biochar to remove oxygen-containing functional groups (-OH, C-O bonds) and enhanced its aromaticity to stabilize carbon skeletons. Sodium silicate modification successfully grafted silanol groups and Si-O bonds onto biochar surfaces, raised hydroxyl content, and retained the intact aromatic carbon skeleton. Modified biochar contained richer oxygen-containing functional groups and underwent dramatic shifts in surface chemical properties.

### 2.2. Nitrogen Leaching and Delayed Release System Characteristics of Biochar-Based Fertilizers

**Figure 3:**
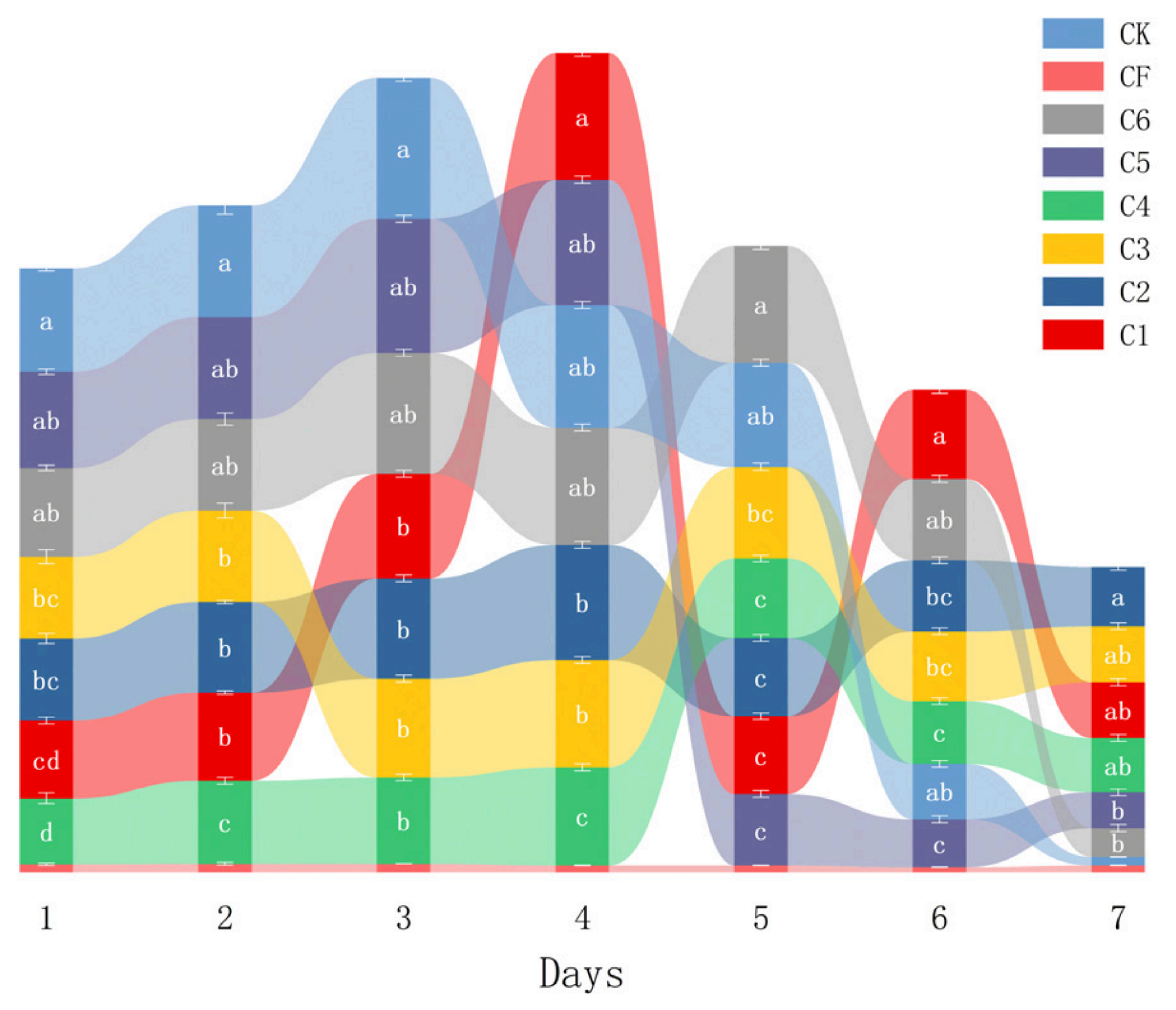
Cumulative nitrogen leaching from different biochar-based fertilizers during soil column incubation. Different small letters indicate a significant difference among groups at *p* < 0.05 based on one-way ANOVA followed by Duncan’s multiple range test.

Figure 3 shows that biochar-based fertilizers effectively slow nutrient release to achieve controlled fertilizer delivery. The nitrogen leaching flux of all treatments followed a unimodal dynamic pattern that initially increased and then declined, although distinct differences existed in nutrient release rhythms among fertilizer systems. The nitrogen leaching peaks for the sole chemical fertilizer treatment (CF) and the 1:4 biochar-to-fertilizer ratio groups (C5, C6) appeared on the 3rd day of leaching, accompanied by substantial short-term nitrogen release. In contrast, treatments with higher biochar proportions at ratios of 1:2 and 1:3 (C1, C2, C3, C4) exhibited delayed nitrogen leaching peaks until the 4th day, confirming that biochar carriers can slow nitrogen release. By the 7th day of the leaching assay, nearly all applied nitrogen had leached out under the CF and C6 treatments, indicating their poor nitrogen retention capacity. For the unmodified biochar-based fertilizer C3 (1:3 ratio), cumulative leached nitrogen accounted for 92% of the total applied nitrogen, suggesting that even the best-performing pristine biochar formulation retained only a small fraction of nitrogen under these conditions. Under identical biochar-fertilizer mixing ratios, silicon-modified biochar-based fertilizers produced lower total nitrogen leaching losses than their pristine biochar counterparts, indicating that silicon functionalization further enhances soil nutrient retention potential.

### 2.3. Effects of Different Fertilization Treatments on Soil Physicochemical Properties and Enzyme Activities

#### 2.3.1. Soil Physicochemical Properties

**Figure 4:**
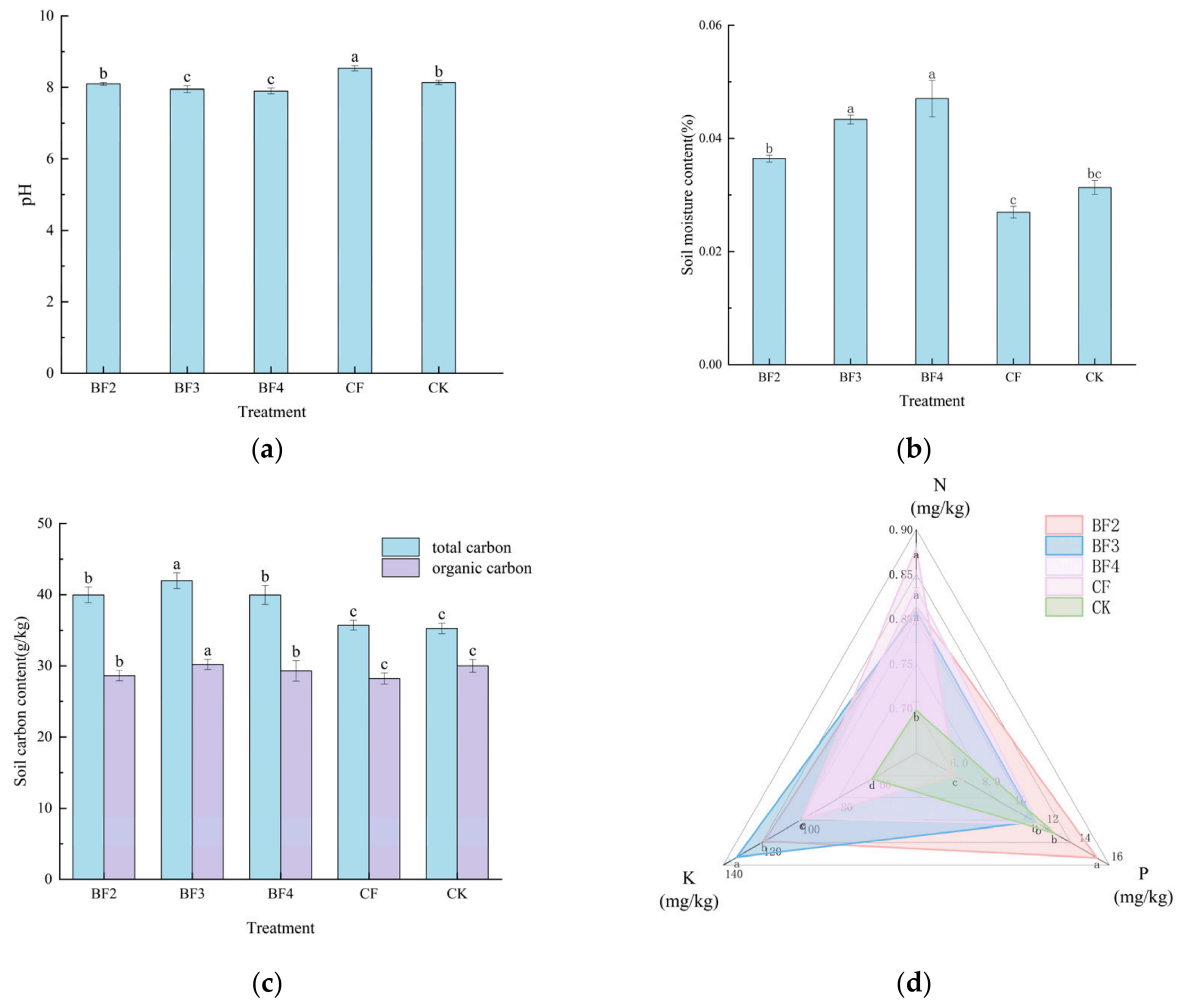
Effects of biochar-based fertilizers with different biochar–fertilizer ratios on soil physicochemical properties. (**a**) Soil pH; (**b**) Soil water content; (**c**) Total carbon and available phosphorus content; (**d**) Available potassium content. CK: no fertilizer control; CF: single chemical fertilizer; BF2/BF3/BF4: silicon-modified biochar-based fertilizers with ratios of 1:2, 1:3, 1:4. The same applies below. Different small letters indicate a significant difference among groups at *p* < 0.05 based on one-way ANOVA followed by Duncan’s multiple range test.

Figure 4 illustrates the influences of biochar-based fertilizers on soil physicochemical indicators. All biochar-based fertilizer treatments recorded higher total carbon, organic carbon, total nitrogen, available phosphorus and available potassium contents yet lower soil water content than the CF treatment. Soil pH declined as the biochar proportion in biochar-based fertilizers increased. Relative to CF, biochar-based fertilizer treatments significantly reduced soil pH by 0.45–0.64 units. Compared with CF, BF2, BF3, and BF4 increased soil water content by 38.46%, 65.38%, and 80.76%, respectively. Urease activity in BF2, BF3, and BF4 rose by 37.93%, 10.50% and 1.44% relative to CF, whereas sucrase activity decreased by 15.01%, 24.16% and 29.61%. No significant differences in soil organic carbon and total nitrogen contents existed among biochar-based fertilizer treatments with different biochar ratios. In comparison with CF, BF2, BF3, and BF4 significantly raised soil total carbon and available phosphorus by 11.87–137.21% and 17.46–77.11%. In addition, BF2 and BF3 treatments markedly increased soil available potassium by 19.89% and 34.16% relative to CF.

#### 2.3.2. Soil Enzyme Activity Characteristics

**Figure 5:**
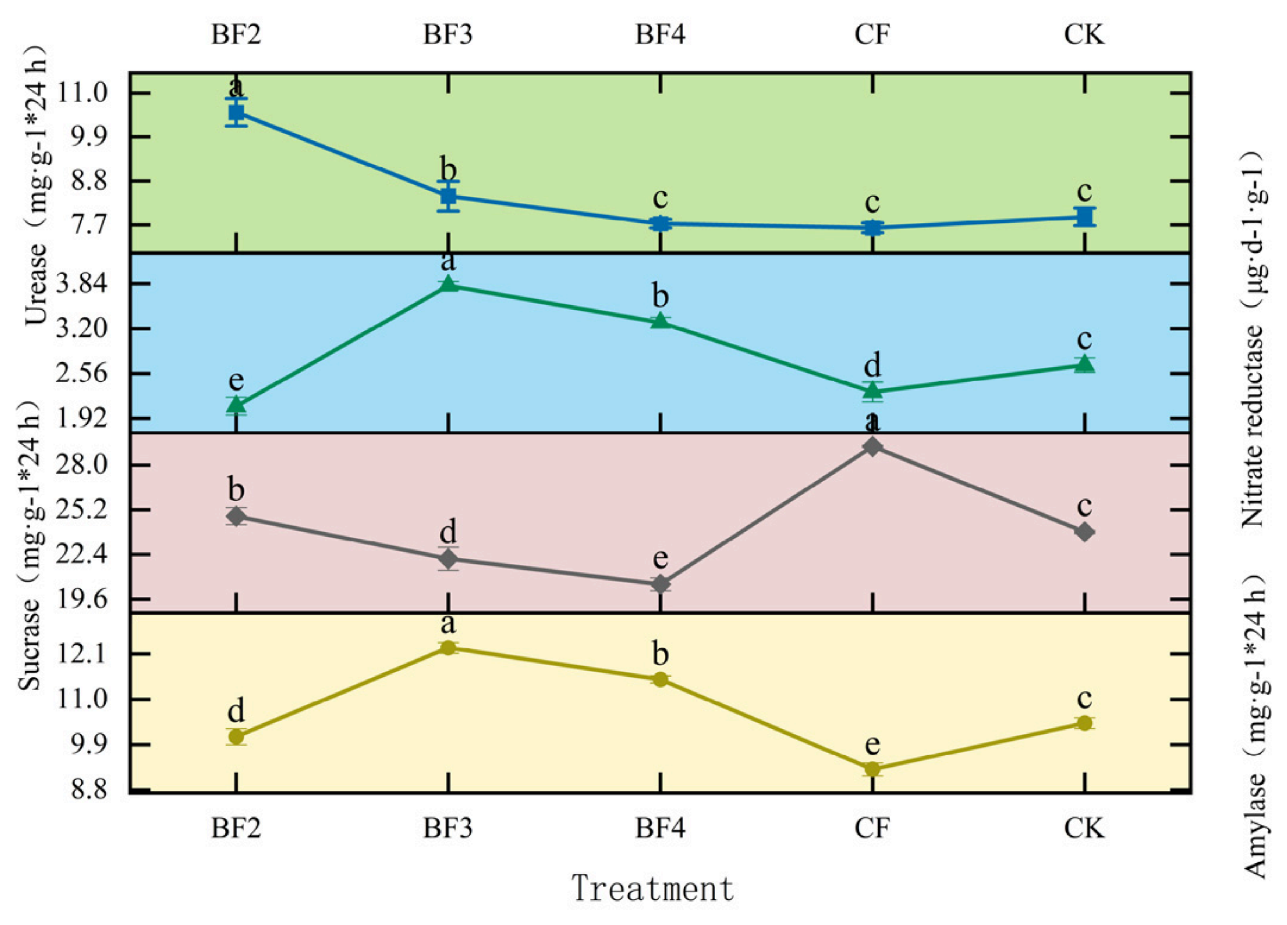
Activities of carbon and nitrogen cycling-related soil enzymes under different fertilization treatments, including urease, nitrate reductase and sucrase. Different small letters indicate a significant difference among groups at *p* < 0.05 based on one-way ANOVA followed by Duncan’s multiple range test.

Figure 5 displayed the variations in soil enzymes related to carbon and nitrogen cycling under different fertilization regimes. BF2 possessed significantly higher urease activity than all other treatments, followed by BF3, BF4, CF and CK showed the lowest urease activity with no statistical differences between them. BF3 and BF4 exhibited remarkably elevated nitrate reductase activity, which increased by 67.84% and 44.49% relative to CF. Sucrase activity declined as the biochar proportion in biochar-based fertilizers increased, and CF obtained the maximum sucrase activity. Amylase activity of BF2, BF3 and BF4 increased by 8.49%, 31.72% and 23.44% compared with CF.

### 2.4. Effects of Fertilization Treatments on Foxtail Millet Growth Traits

**Figure 6:**
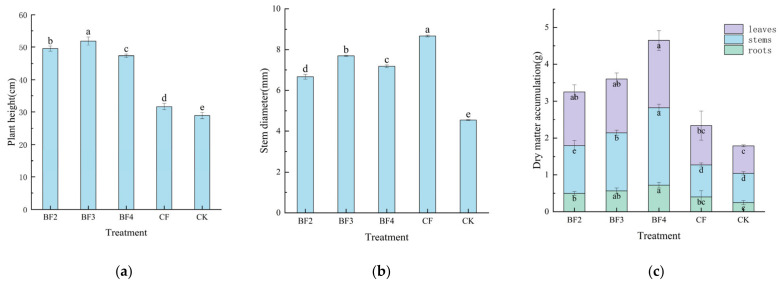
Growth traits of foxtail millet under different biochar-based fertilizer treatments. (**a**) Plant height; (**b**) stem diameter; (**c**) root, stem and leaf dry matter accumulation. Different small letters indicate a significant difference among groups at *p* < 0.05 based on one-way ANOVA followed by Duncan’s multiple range test.

Figure 6 indicates that all biochar-based fertilizer treatments significantly improved foxtail millet plant height and dry matter accumulation in roots, stems and leaves relative to the single chemical fertilizer treatment. The ranking of foxtail millet plant height across treatments was BF3 > BF2 > BF4 > CF > CK. Compared with CF, BF2, BF3 and BF4 boosted plant height by 56.24%, 63.72% and 49.43%. CF yielded significantly thicker stems than all biochar-based fertilizer treatments, with stem diameters 30.09%, 12.83% and 20.90% larger than BF2, BF3 and BF4, respectively; among biochar-based fertilizer groups, BF3 produced the thickest millet stems. Dry matter accumulation in roots, stems and leaves under biochar-based fertilizer treatments rose by 38.89–81.56%, 54.41–101.73% and 99.06–161.23% relative to CF and CK, while CF increased total biomass by 31.23% compared with CK.

## 3. Discussion

### 3.1. Regulatory Effects of Pyrolysis Temperature and Sodium Silicate Modification on Biochar Structural Properties

The physicochemical traits of biochar are governed jointly by feedstock type and pyrolysis temperature, which directly determine its potential as a soil amendment and fertilizer carrier [23]. This study observed that the C/N ratio of maize stover biochar increased with pyrolysis temperature, while hydrogen and oxygen contents underwent progressive decomposition—a trend consistent with prior investigations on lignocellulosic pyrolysis [24,25]. High-temperature conditions remove oxygen-containing functional groups from biochar surfaces and stabilize its carbon framework [26]. The present experiment identified 500 °C as the optimal pyrolysis threshold, under which biochar attained its maximum specific surface area and fully developed pore architecture. When temperatures exceeded 550 °C, pore collapse occurred, accompanied by a sharp reduction in specific surface area and weakened adsorption capacity. These observations validate the existence of a critical thermal threshold for biochar production [27]; excessive heating destroys porous frameworks and diminishes its applicability as a fertilizer carrier.

Sodium silicate functionalization further refines the physical architecture and surface chemical properties of biochar. The silicon-modified biochar produced at 500 °C exhibited a specific surface area of 262.79 m^2^/g. Fourier-transform infrared spectroscopy (FTIR) analysis indicated successful grafting of silanol groups and Si-O linkages onto the material surface without disrupting the inherent aromatic carbon skeleton. Scanning electron microscopy revealed that biochar undergoes changes in surface morphology and exhibits increased surface roughness, substantially increasing the number of effective adsorption sites [28]. Silicon-bearing functional groups and hierarchical pores boost the hydrophilicity and nutrient retention capacity of biochar, rendering it an excellent slow-release fertilizer matrix [29].

### 3.2. Soil Improvement and Nutrient Slow Release of Silicon-Modified Biochar-Based Fertilizers

Soil column leaching experiments confirmed that biochar immobilizes nutrients through its porous structure and surface functional groups, thereby reducing leaching losses and sustaining slow nutrient release [30]. Silicate modification further enhances nutrient retention and prolongs the release period, ensuring a stable nutrient supply throughout the cropping season. Biochar-based fertilizers also reduce soil pH while increasing carbon stocks and available nitrogen, phosphorus, and potassium. These improvements are attributable to the strong acid-base buffering capacity of silicate-modified biochar-based fertilizers, which stems from proton exchange by surface oxygen-containing functional groups, pH buffering by silanol groups, and enhanced cation exchange capacity [31,32]. The developed pore structure of biochar improves soil water retention, with the magnitude of improvement increasing at higher application rates.

Soil enzymes involved in carbon and nitrogen turnover are key indicators of nutrient transformation and microbial metabolic activity. Biochar-based fertilizer treatments enhanced the activities of urease, nitrate reductase, and amylase; exogenous organic carbon can serve as a source of substrates for soil enzyme synthesis, thereby altering the secretion levels of enzymes involved in soil carbon-nitrogen transformation [33]. However, sucrase activity was highest under the CF treatment, a result contrasting with most previous biochar studies. This discrepancy can be attributed to substrate availability. Sucrase activity depends strongly on soluble inorganic nitrogen and phosphorus concentrations in soil. Without biochar-mediated nutrient immobilization, the CF treatment maintained higher transient concentrations of labile mineral nutrients in soil solution, which rapidly stimulated the growth of sucrase-producing microorganisms [34]. In contrast, biochar immobilizes mineral nutrients, thereby lowering dissolved nutrient concentrations and indirectly reducing sucrase activity [35]. This differential response suggests that biochar-based fertilizers selectively modulate carbon-metabolizing enzymes rather than universally enhancing all carbon-cycling enzyme activities.

### 3.3. Growth-Promoting Mechanisms of Biochar-Based Fertilizers on Foxtail Millet

Biochar-based fertilizers facilitate crop biomass accumulation by ameliorating rhizosphere microhabitats and delivering nutrients in a steady manner [36]. In the present trial, all biochar-amended fertilization treatments yielded significantly greater plant height and dry matter accumulation of foxtail millet compared with the sole chemical fertilizer (CF) and blank control groups [37,38]. Nevertheless, a divergent vegetative response was observed: millet stems under CF treatment were markedly thicker than those receiving biochar-based fertilizers.

This discrepancy arises from the rapid burst of labile nutrients under chemical-only fertilization, which supplies abundant mineral nutrition to support radial stem expansion within a short period. In contrast, the slow-release mechanism of biochar-based fertilizers prioritizes nutrient allocation to vertical shoot elongation, leading to asynchronous growth between plant height and stem diameter. Additionally, excessive biochar incorporation induces transient disturbances to rhizosphere conditions, thereby constraining lateral stem development. Similar morphological differentiation induced by biochar application rates has been documented in pot experiments involving quinoa and sorghum. This observation underscores that crop performance does not increase linearly with biochar dosage; an appropriate biochar-to-fertilizer ratio constitutes the core prerequisite for maximizing the yield-promoting benefits of biochar-based fertilizers.

## 4. Materials and Methods

### 4.1. Test Materials

Test maize stover and topsoil (0–20 cm) were collected from the Shenfeng Experimental Base of Shanxi Agricultural University in Taigu District, Jinzhong City, Shanxi Province. Maize stover was cut into 2 cm segments and oven-dried to constant weight for later use. Soil samples were air-dried and sieved through a 20-mesh screen. The basic soil fertility indicators were as follows: organic matter 10.69 g/kg, total nitrogen 0.71 g/kg, alkali-hydrolyzable nitrogen 48.25 mg/kg, available phosphorus 3.52 mg/kg, available potassium 174.96 mg/kg, and soil pH 8.52. The tested foxtail millet cultivar was Jingu 21.

### 4.2. Test Methods

#### 4.2.1. Biochar Production and Sodium Silicate Modification

Dried straw samples were placed in crucibles and transferred to a muffle furnace. Biochar was produced at five gradient pyrolysis temperatures (400, 450, 500, 550, 600 °C). The furnace heated up to the target temperature at a rate of 10 °C/min, maintained the temperature for 4 h, and cooled naturally to obtain crude biochar (BC). The prepared biochar was ground and sieved through a 60-mesh screen for standby application.

Raw biochar was mixed with 0.2 mol/L sodium silicate solution (analytical grade, Na_2_SiO_4_·9H_2_O) at a mass ratio of 1:10 and stirred evenly. The mixture was sealed with plastic wrap and placed in a cool environment for 24 h. After oven-drying and grinding through a 60-mesh sieve, the biochar was rinsed with distilled water 3–4 times until the filtrate turned neutral to remove residual surface salts. The sample was dried at a constant temperature of 80 °C for 24 h to obtain silicon-modified biochar (BCSi), which was stored in a desiccator.

#### 4.2.2. Characterization of Raw and Modified Biochar

A fully automatic specific surface area and porosity analyzer (Micromeritics ASAP 2460, Micromeritics Instrument Corporation, Norcross, GA, USA) measures the specific surface area and pore size distribution of biochar samples. A scanning electron microscope (JSM-7610F JEOL Ltd., Tokyo, Japan) observes surface morphology. Fourier-transform infrared spectroscopy (Thermo Fisher Scientific Nicolet iS20, Madison, WI, USA) characterizes surface functional groups. An elemental analyzer (Elemantar Vario EL cube, Langenselbold, Germany) detects the elemental composition of biochar surfaces. All physical and chemical characterizations were conducted on independent biochar samples prepared by the same batch of pyrolysis.

#### 4.2.3. Biochar-Based Fertilizer Production, Soil Column Leaching Test and Pot Experiment Design

Raw and silicon-modified maize stover biochar produced at 500 °C served as carriers for biochar-based fertilizers. Urea, diammonium phosphate, potassium chloride and bentonite were mixed with an appropriate amount of clean water. In accordance with the national standard GB/T 18877-2020, a disk granulator was used to produce biochar-based fertilizers at three biochar-fertilizer mass ratios (1:2, 1:3, 1:4). The nutrient content per kilogram of each fertilizer is shown in Table 3.

Soil column leaching experiments were conducted using self-fabricated PVC column devices. A filter screen was laid and fixed at the bottom of each soil column. Each column was filled with 350 g of air-dried test soil. The test fertilizers were evenly spread on the top of the soil layer, followed by covering with another 150 g of air-dried soil, which created a 4.5 cm fertilizer burial depth. This setup simulated the actual field fertilization pattern and effectively reduced volatilization loss of surface soil nutrients. Deionized water was used to simulate natural precipitation and irrigation during leaching. On the first day, 250 mL of deionized water was added at once to form the leaching layer, and the leachate was collected for total nitrogen determination. For the subsequent 7 consecutive days, 100 mL of deionized water was quantitatively supplied daily, and leachate samples were collected at fixed intervals for subsequent analysis. A total of eight treatments were established in this experiment, including pristine biochar-based fertilizer, silicon-modified biochar-based fertilizer, chemical fertilizer alone, and blank control, with three replicates for each treatment. The total nitrogen concentration of the leachate was determined via the Kjeldahl nitrogen analyzer.

Pot experiments were carried out in the greenhouse at the Agronomy Experimental Station of Shanxi Agricultural University. Five experimental treatments were set up, namely no-fertilizer blank control (CK), sole chemical fertilizer (CF), biochar-fertilizer mass ratios of 1:2 (BF2), 1:3 (BF3), and 1:4 (BF4), with three replicates per treatment. Each pot was filled with 1 kg of air-dried soil fully homogenized with the corresponding fertilizer. Six seeding holes of millet cultivar Jingu 21 were sown in each pot at a sowing depth of 3 cm. Soil moisture was maintained at an appropriate level throughout the millet growth period. Plant and soil samples were collected 45 days after sowing to measure relevant physiological and chemical indices.

#### 4.2.4. Determination of Soil Indicators

The core cutting ring method was applied to determine soil water content. Soil pH was measured via a pH meter with a soil-water mixing ratio of 1:2.5. A carbon-nitrogen analyzer determines soil total carbon and organic carbon. A continuous flow analyzer tests total nitrogen in digested soil samples. The molybdenum-antimony anti-colorimetric method measures soil available phosphorus. A flame photometer detects soil available potassium. The phenol disulfonic acid colorimetric method assays nitrate reductase activity. The phenol-sodium hypochlorite colorimetric method determines urease activity.

#### 4.2.5. Determination of Foxtail Millet Growth Indicators

Foxtail millet seedlings were excavated 45 days after sowing. A straightedge and vernier caliper were used separately to measure plant height and stem diameter. Plant samples were placed in an oven for deactivation of enzymes at 105 °C for 30 min, dried to constant weight at 75 °C, and weighed with an electronic balance to record total dry biomass.

### 4.3. Data Processing and Statistical Analysis

Excel 2019 was used for basic data sorting and statistics. SPSS 2023 was adopted to conduct one-way analysis of variance (ANOVA) and least significant difference (LSD) multiple comparisons at a significance level of *p* < 0.05. Omnic 9 (2016) software processes Fourier-transform infrared spectral data and draws spectrograms. Origin 2022 generates all experimental figures.

## 5. Conclusions

This experiment investigated the regulatory effects of different pyrolysis temperatures on the structural properties of biochar, as well as the variation characteristics of foxtail millet growth, soil physicochemical properties and soil enzyme activities under the application of biochar-based fertilizers with different biochar-fertilizer ratios. The main findings are as follows: An appropriate pyrolysis temperature combined with sodium silicate modification can effectively enhance the nutrient adsorption and retention capacity of biochar. Moreover, an appropriate blend of biochar-based fertilizers can effectively regulate soil physicochemical properties, markedly reduce soil pH, improve soil water retention capacity and enhance soil nutrient storage and supply capacity, thereby significantly promoting the growth of foxtail millet plant height and stem diameter, as well as plant dry matter accumulation. Based on the comprehensive experimental data, the BF3 biochar-based fertilizer application scheme has the best overall effect and can provide a theoretical basis for the development of special biochar-based fertilizers for grains. It also provides a technical solution for the cost reduction and efficiency improvement of the sustainable development of grains.

However, this study has certain limitations. Firstly, only SEM and FTIR were used for qualitative confirmation of silicon element loading, and quantitative silicon loading content was lacking using XRF and ICP-OES methods. Secondly, only short-term pot experiments were carried out in the present research. Long-term field-located trials are still required to verify the long-term fertilizer efficiency and soil ecological effects of raw and silicon-modified biochar under field conditions, as well as the stability evolution of silicon-modified biochar in soil.

## Figures and Tables

**Table 1:** Elemental Composition and pH of Biochar and Modified Biochar at Different Pyrolysis Temperatures.

Treatment	C(%)	N(%)	C/N	H(%)	S(%)	O(%)	pH Value
BC-400	65.04 ± 1.07 b	1.96 ± 0.06 a	33.18	3.24 ± 0.06 a	0.16 ± 0.02 a	20.27 ± 0.07 a	10.02 ± 0.03 d
BC-450	67.16 ± 1.40 b	1.90 ± 0.05 a	35.35	3.03 ± 0.07 b	0.12 ± 0.01 b	17.51 ± 0.26 c	10.08 ± 0.01 c
BC-500	69.34 ± 0.89 a	1.96 ± 0.09 a	35.38	2.75 ± 0.16 c	0.13 ± 0.02 b	15.56 ± 0.16 e	10.13 ± 0.01 b
BC-550	69.20 ± 0.70 a	1.75 ± 0.11 b	39.54	2.53 ± 0.05 d	0.12 ± 0.01 b	14.20 ± 0.10 g	10.15 ± 0.01 b
BC-600	70.22 ± 1.22 a	1.70 ± 0.05 bc	41.31	2.17 ± 0.09 e	0.09 ± 0.02 c	13.99 ± 0.20 g	10.23 ± 0.01 a
BCSi-400	58.28 ± 0.73 d	1.61 ± 0.03 cd	36.20	3.01 ± 0.08 b	0.06 ± 0.03 d	20.02 ± 0.18 a	9.43 ± 0.01 h
BCSi-450	60.97 ± 1.52 c	1.66 ± 0.09 bc	36.73	2.94 ± 0.14 b	0.06 ± 0.01 d	19.47 ± 0.14 b	9.48 ± 0.03 g
BCSi-500	65.32 ± 0.92 b	1.78 ± 0.06 b	36.70	2.66 ± 0.07 cd	0.06 ± 0.01 d	16.59 ± 0.23 d	9.51 ± 0.02 g
BCSi-550	66.19 ± 1.13 b	1.77 ± 0.03 b	37.40	2.54 ± 0.07 d	0.06 ± 0.01 d	15.55 ± 0.08 e	9.62 ± 0.01 f
BCSi-600	66.97 ± 1.39 b	1.52 ± 0.03 d	44.06	2.20 ± 0.19 e	0.05 ± 0.02 d	14.91 ± 0.09 f	9.73 ± 0.02 e

Values are presented as mean ± standard deviation; different lowercase letters within the same column indicate significant differences at *p* < 0.05.

**Table 2:** Pore Structures of Biochar and Modified Biochar at Different Pyrolysis Temperatures.

Biochar Types	BET (m^2^/g)	Pore Diameter (nm)	Total Pore Volume (cm^3^/g)
BC-400	6.10	4.23	0.01
BC-450	149.29	2.34	0.02
BC-500	221.79	1.84	0.10
BC-550	74.77	2.01	0.04
BC-600	38.96	4.00	0.01
BCSi-400	7.63	1.63	0.06
BCSi-450	215.70	1.87	0.10
BCSi-500	262.79	1.81	0.12
BCSi-550	209.95	1.67	0.09
BCSi-600	60.70	2.32	0.04

**Table 3:** Nitrogen, phosphorus, potassium, and carbon contents of biochar-based fertilizers (g kg^−1^).

Types of Fertilizers	Charcoal Fertilizer Ratio	N	*p*	K	C
C1	Biochar-Based Fertilizer 1:2	0.14	0.09	0.12	0.18
C2	Silica-Modified Biochar-Based Fertilizer 1:2	0.14	0.09	0.12	0.18
C3	Biochar-Based Fertilizer 1:3	0.14	0.09	0.12	0.12
C4	Silica-Modified Biochar-Based Fertilizer 1:3	0.14	0.09	0.12	0.12
C5	Biochar-Based Fertilizer 1:4	0.14	0.09	0.12	0.09
C6	Silica-Modified Biochar-Based Fertilizer 1:4	0.14	0.09	0.12	0.09
CF	Fertilizer treatment	0.14	0.09	0.12	0.00
CK	Blank Control	0.00	0.00	0.00	0.00

C1, C3, C5: unmodified biochar fertilizers at ratios 1:2, 1:3, 1:4; C2, C4, C6: silica-modified counterparts with matching ratios; CF: pure chemical fertilizer; CK: no-fertilizer blank control. Values denote element mass per kilogram of fertilizer.

## Data Availability

All data generated or analyzed in this study are included in this published article. For further information, please contact the corresponding author.
